# The effects of dictatorship on health: the case of Turkmenistan

**DOI:** 10.1186/1741-7015-5-21

**Published:** 2007-07-30

**Authors:** Bernd Rechel, Martin McKee

**Affiliations:** 1 European Centre on Health of Societies in Transition, London School of Hygiene and Tropical Medicine, Keppel Street, London, UK

## Abstract

**Background:**

There is a health crisis in Turkmenistan similar to, but more severe than, in other Central Asian countries. This paper asks whether the health crisis in Turkmenistan is attributable to the consequences of the dictatorship under president Niyazov, who died in 2006.

**Methods:**

The basis for this paper was a series of semi-structured in-depth interviews with key informants complemented by an iterative search of internet sites, initially published as a report in April 2005, and subsequently updated with feedback on the report as well as a comprehensive search of secondary information sources and databases.

**Results:**

This paper describes in depth three areas in which the dictatorship in Turkmenistan had a negative impact on population health: the regime's policy of secrecy and denial, which sees the "solution" to health care problems in concealment rather than prevention; its complicity in the trafficking of drugs from Afghanistan; and the neglect of its health care system.

**Conclusion:**

The paper concludes that dictatorship has contributed to the health crisis facing Turkmenistan. One of the first tests of the new regime will be whether it can address this crisis.

## Background

Turkmenistan is a country in Central Asia that, initially reluctantly, became independent as a consequence of the collapse of the former Soviet Union in 1991. The energy-rich and largely desert country, covering an area approximately as large as Spain, has a population of about 5 million people, the majority of which lives in rural areas. In terms of human rights, Turkmenistan provides one of the worst examples of post-Soviet development. The country has become increasingly totalitarian and its human rights violations have been condemned by the United Nations (UN), the Organisation for Security and Cooperation in Europe (OSCE), and the European Parliament. The impact of the authoritarian rule by former president Saparmurat Niyazov on the health of the country's population, however, has so far attracted little international attention.

### Political context

Turkmenistan is a one-party state, which was dominated for 21 years by president Saparmurat Niyazov, until his unexpected death, on 21 December 2006, from heart failure. Niyazov's rule began in 1985 when he became party secretary of the then Turkmen Soviet Socialist Republic. He emerged from the collapse of the Soviet Union as president of the now independent Turkmenistan, where he established one of the most oppressive regimes of any former Soviet republic. He surrounded himself with a bizarre personality cult and became president for life in 1999. On 11 February 2007, Gurbanguly Berdymukhammedov, the former Minister of Health, was, as had been widely predicted, elected as the new president of the country. In the *pro forma *elections, only candidates of the ruling party were allowed, and Berdymukhammedov allegedly gained 89% of the vote, with a participation of 98.65% of voters [1,2].

Turkmenistan has been described as one of the world's most repressive and closed countries [3]. There are no opposition parties or independent trade unions and the government controls the media and censors all newspapers and access to the internet. According to the Annual Worldwide Press Freedom Index, created by Reporters Without Borders, in 2006, Turkmenistan ranked among the two worst violators of press freedoms among 168 countries, surpassed only by North Korea [4]. Civil society activists have faced persecution and imprisonment and the government prevents all but a few non-governmental organisations from working [5]. Prisoners are subject to torture, routine beatings, food deprivation and overcrowding, often leading to death [6]. There have also been politically motivated beatings and incarceration in psychiatric facilities to silence perceived dissidents [3].

The political system of the country has also left its mark on the educational system, which has markedly deteriorated in recent years. In 2002, mandatory education was reduced from 10 to 9 years, while the number of students in higher education has dropped dramatically.

### Socioeconomic context

Although Turkmenistan has one of the largest gas and oil reserves in the world and is one of the top ten cotton producers, the economic potential of the country has not benefited the population. While Niyazov reportedly had control over a US$3 billion trust fund held by Deutsche Bank in Frankfurt, much of the population seems to live in poverty, despite the fact that gas, water, electricity and salt are provided free of charge. In 2003, 58% of the population were estimated to live below the national poverty line [7]. The official unemployment rate is zero, as the state guarantees employment for every Turkmen citizen. However, surveys indicate that *de facto *unemployment is growing, especially among youth [8]. Only 55% of the population is believed to have access to safe drinking water; a figure declining to 24% in rural areas [9], and 8% of the population was estimated to be undernourished in 2003 [10].

## Materials and methods

This paper updates and extends the findings of a report on human rights and health in Turkmenistan that we published in April 2005 [11]. The original report was based on a systematic review of evidence available on the internet and in reports from international and non-governmental organisations, and on information obtained from telephone interviews with key informants, comprising Turkmen exiles, staff of international and non-governmental agencies, and staff of foreign embassies in Turkmenistan.

This paper focuses on three key areas in which the dictatorship has had an impact on health in Turkmenistan: a policy of secrecy and denial; complicity in drug trafficking; and the neglect of the health sector. When updating the findings of our earlier report, we made a comprehensive search of publicly available sources in February 2007. A search was conducted of PubMed/Medline, Google, and Google Scholar, using the word Turkmenistan with a range of health-related terms, with follow up of leads generated. In addition, a search of relevant websites was undertaken, which included the websites of UN agencies, non-governmental organizations, and news agencies (such as EurasiaNet, IRIN News, and the Institute for War and Peace Reporting). The article reflects the situation in Turkmenistan in February 2007.

## Results

Do the profound human rights violations in Turkmenistan have an impact on the health of the people living there? Recent research has shown a surprisingly close association between the extent of political freedom and several measures of population health, after adjusting for economic factors [12]. However, the precise nature of the impact of dictatorship on health is difficult to quantify, as health is influenced by a large number of factors, including the socioeconomic situation, housing, nutrition, drinking water, lifestyle and access to health services.

Health impacts of human rights violations in Turkmenistan are most obvious in the imprisonment, torture and beatings of perceived opponents of the regime, and the incarceration of part of the population in unsanitary and overcrowded penal colonies. As in the Soviet period, psychiatry is being abused for political purposes, with perceived opponents of the regime being confined to psychiatric institutions. The suppression or deportation of religious and ethnic minorities, and the demolition of private homes to make way for grandiose presidential projects also directly impact on health and well-being. The impoverishment of the population, while the leadership is amassing great private fortunes, also has consequences for health, affecting access to the essentials for life, such as nutrition, and to basic health services. There are credible accounts of how impoverishment and lack of opportunities are leading to increasing sex work, domestic violence and drug use. The suppression of civil society, the growing isolation of the country and the general climate of fear, repression and corruption are likely to contribute to deteriorating population health, as are the violations of the right to education [11].

This paper explores in more detail three ways in which the dictatorship in Turkmenistan has impacted negatively on the health of the population living there. It first describes how the regime has pursued a policy of secrecy and denial, which sees the "solution" to health care problems in concealment rather than prevention. The paper then explores the complicity of the regime in drug trafficking, which has led to widespread drug use in Turkmenistan and also impacts on the health and well-being of people in the destination countries of the international drug trade. The paper concludes by describing how the regime has neglected the health care system in recent years and seeks to gauge the extent of the health crisis in the country.

### A policy of secrecy and denial

A state's failure to recognize or acknowledge health problems can result in severe human rights violations [13]. While the right to "seek, receive and impart information and ideas through any media and regardless of frontiers" [14] has been enshrined in Article 19 of the Universal Declaration of Human Rights, in Turkmenistan, violations of the right to information have become common. In our 2005 report we have described these violations as a policy of "secrecy and denial" [11]. The United Nations Resident Coordinator in Turkmenistan noted in a similar vein in 2006 that "the lack of reliable data still remains a major challenge for UN agencies" [15], while the most recent country report of the US Department of State found that "most statistical data was considered a state secret" [5].

Taking account of new developments, however, a rather complex picture emerges. On the one hand, Turkmenistan has failed to report some health indicators to the international community. In 2000 the Turkmen government stopped reporting some health indicators to the World Health Organization (WHO), with the most recent data on life expectancy for example relating to 1998. The country has also failed to report credible data on the incidence of HIV, tried to cover up an outbreak of plague, and issued an unofficial ban on diagnosing communicable diseases.

On the other hand, however, Turkmenistan has continued to publish some health data, including the incidence of tuberculosis. Turkmenistan has also tried to gain external assistance for the fight against some communicable diseases, which implicitly acknowledges that they might be a problem for the country. The Turkmen government has submitted an application to the Global Fund to Fight AIDS, Tuberculosis and Malaria, and has developed an array of activities against the spread of avian influenza (which, according to official data, has yet to be detected in Turkmenistan). In January 2007, Turkmenistan has also begun using the international live birth definition recommended by WHO, which will help to establish more accurate data on infant mortality. Adoption of the WHO live birth definition was one of the policy recommendations of our 2005 report and international agencies, including the United States Agency for International Development (USAID), the United Nations Children's Fund (UNICEF), the United Nations Population Fund (UNFPA), and the Joint United Nations Programme on HIV/AIDS (UNAIDS), have advocated for this policy change since 2002 [16]. There is, however, no independent means of assessing whether the limited data supplied are accurate.

A more general problem, however, is that many health professionals and the general population have no access to information related to health and health care in Turkmenistan or other countries, or to international medical literature. Lack of access to health-related literature or the internet means that the population has very limited access to information about prevention or treatment. The almost complete dominance of the Turkmen language in official contexts, but in which very few medical works are published, contributes to the isolation of the country, although in practice many are unable to speak Turkmen and use Russian instead. We have been told of medical lectures being given in Turkmen, by teachers who could barely speak the language, to students with limited understanding of it, while struggling with medical terms that do not exist in Turkmen. Overall, there is a shortage of training materials and medical knowledge is outdated. In addition, very few health professionals are allowed to take part in international conferences.

#### The reported ban on diagnosing infectious diseases

Article 12 of the International Covenant on Economic, Social and Cultural Rights obliges member states to prevent, treat and control "epidemic, endemic, occupational and other diseases" [17]. In Turkmenistan, the regime has at times decided to deny the existence of health problems instead of addressing them. An unofficial ban on diagnosing infectious diseases was issued in 2004 by the Turkmen Ministry of Health and Medical Industry. According to the Watan opposition site and the Turkmenistan Helsinki Foundation, Turkmen health care officials have issued secret instructions banning, from 1 May 2004, any mention of diseases such as tuberculosis, measles, dysentery, cholera and hepatitis, which may lead to epidemics [18-20], in order to "assure the international community of the absolute well-being and the complete non-existence of any contagious diseases and problems with medication and treatment in Turkmenistan" [19,20].

Doctors were reportedly prohibited from mentioning these diseases in any documents, including death certificates [20,21]. However, despite these reported instructions, the existence of tuberculosis has been acknowledged by the government and data on tuberculosis incidence have been reported in government publications and to WHO.

#### Plague

A plague outbreak in Turkmenistan was reported in June 2004 by a Russian newspaper and by Gundogar, an opposition website. Various sources have claimed that the outbreak was responsible for up to 10 deaths [22]. The Turkmenistan Helsinki Initiative reported that plague victims were taken to a hospital outside Ashgabat, where they were secretly treated. The district hospital was cleared of all other patients, and surrounded by soldiers and special services employees [23]. Health workers were reportedly told to keep silent about the outbreak. According to a clinic worker, "If any of us said that there was plague in the city, they would be arrested and charged with revealing state secrets" [23]. Doctors were ordered to diagnose "food poisoning" as the cause of death [23].

The government responded to the outbreak by instituting border controls "to prevent disease from entering Turkmenistan from neighbouring states" [24]. Officials have denied any outbreak of plague. The Anti-Epidemic Emergency Commission claimed that "the epidemiological situation on the territory of Turkmenistan is safe. There are no cases of dangerous diseases" [19]. International experts worry that the health care system will be unable to cope with a plague epidemic. According to an American epidemiologist, "the expertise on the local level to diagnose anything is limited" [19].

#### HIV/AIDS

The Turkmen government does not publish credible data about the incidence of HIV/AIDS in Turkmenistan and denies that there have been new HIV infections in recent years. According to UNAIDS, by 2004 only two cases of HIV/AIDS had ever been reported in Turkmenistan [25], while according to the data reported to UNICEF by the Turkmen Statistical Office, five newly registered HIV/AIDS cases had been reported by 2004, with not a single case reported since 2000 [26].

**Figure 1:**
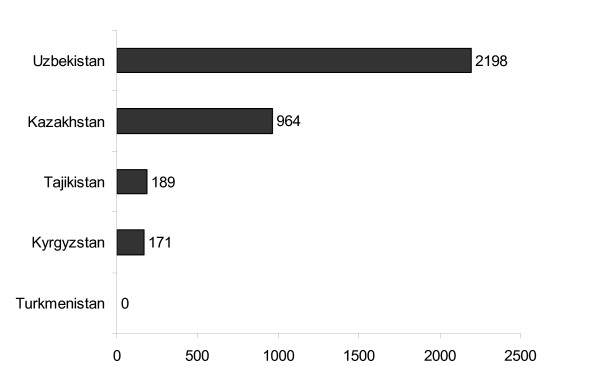
Number of new infections with HIV in the countries of Central Asia in 2005. Source: WHO Health for All Database [78].

While there are a number of concerns about officially recorded data on HIV in most countries of the former Soviet Union [27], the data published by Turkmenistan become even more questionable when put in the regional context of Central Asia (Figure 1).

As the other countries in Central Asia, Turkmenistan has considerable potential for an HIV/AIDS epidemic, due to widespread injecting drug use, sex work and high rates of sexually transmitted infections [28]. Anonymous testing for HIV/AIDS is reported as being unavailable and there do not seem to be support services for people living with HIV/AIDS [21]. The absence of civil society organisations means that the needs of vulnerable groups are generally ignored.

In its 2006 epidemiological fact sheet, UNAIDS estimated that there had been up to 1,000 HIV/AIDS cases in the country in 2005, equivalent to less than 0.2% of the population [25]. According to an unofficial source in the Ministry of Health and Medical Industry, there were more than 300 confirmed cases of HIV infection in Ashgabat alone, with the real figure considerably higher [21]. The Turkmen State News Service, however, reported in 2001 that "AIDS is not a problem in Turkmenistan due to the success of the governmental anti-AIDS measures" [29].

The government formulated a five-year National Programme on HIV/AIDS/STI Prevention in 1999 (1999–2003), followed by a new national programme for 2005–2010, and in 2001 adopted a law "On preventing HIV infection". A National AIDS Centre was established, with the main office in Ashgabat and branches in all five velayats (regions). In 2004, the country had 27 diagnostic laboratories that could perform HIV serological testing [29]. The country has also established a formal Inter-Agency Coordination Committee and launched information campaigns, targeting youth, sex workers and prisoners [30]. UN agencies, the British embassy and USAID support the government of Turkmenistan in the implementation of the national programme on prevention of HIV/AIDS and sexually transmitted infections for the period 2005–2010. An application to the Global Fund to Fight AIDS, Tuberculosis and Malaria has now been submitted, although it remains to be seen whether Turkmenistan could comply with the requirements of the Global Fund for independent verification of expenditure and activity. The proposal is aimed at expanding the TB-DOTS (Tuberculosis – Directly Observed Therapy Short-course) programme nationwide, introducing HIV/AIDS prevention activities, and preventing malaria [31]. At present, Turkmenistan is the only Central Asian country not receiving Global Fund support.

#### Tuberculosis

Like the other countries in Central Asia, Turkmenistan faces very high rates of tuberculosis. A DOTS programme was introduced in Dashoguz velayat (the least developed in Turkmenistan) in 1998. Turkmenistan's National TB-DOTS Programme envisages a gradual expansion of the DOTS programme to cover the entire country by 2009, and the country is receiving external assistance from international agencies in doing so [32]. Yet, a World Bank report found in 2004 that "overall, there is still insufficient government commitment to TB control in Turkmenistan" [29].

#### Avian influenza

While cases of avian flu in animals or humans have so far not yet been officially detected in Turkmenistan, carcasses of migratory birds suspected of carrying the Avian flu virus were discovered on Turkmenistan's Caspian Sea coast early in 2006 [33]. This has triggered an array of activities, including the establishment of the "National Epidemiological Commission on Prevention of Avian Influenza", the adoption in January 2006 of a National Plan of Action for the prevention and control of avian influenza and pandemic preparedness, and a funding proposal to the UN country team. A number of capacity building measures have been initiated, with support of WHO, UNICEF, the Food and Agriculture Organization of the United Nations (FAO), and USAID.

### Complicity in drug trafficking

Many observers have concluded that senior members of the Turkmen regime are complicit in the trafficking of drugs from Afghanistan, many of which reach Western Europe via Russia [34]. While widespread corruption, fuelled by low public sector salaries, is a problem in all Central Asian countries [35], many foreign observers believe that narcotics trafficking became a major source of revenue for the Niyazov regime [36]. It has been speculated that, before 11 September 2001, Turkmenistan's cordial relations with the Taliban regime in Afghanistan were based on the profitable drug trade [36]. Many corrupt officials in Turkmenistan are turning a blind eye to the problem or are complicit in drug trafficking [37]. In the regions close to the Afghan border, the majority of men have reportedly been convicted of drug trafficking, although it is possible to be released from prison when paying a bribe and many convicted of drug trafficking are released through the annual prison amnesties [38].

While Turkmenistan had in the past not joined several regional activities aimed at preventing the trafficking of drugs, it has decided to participate in Operation Topaz, which aims to prevent the proliferation of precursor chemicals for the manufacture of heroin [39], and in 2006, Turkmenistan became one of the founders of the joint regional law enforcement body – Central Asian Regional Information and Cooperation Centre, facilitated by the United Nations Office on Drugs and Crime (UNODC) [40].

In February 2006, Turkmenistan's customs and border officials were taking part in training courses on fighting drug trafficking, which were organized by the UNODC, with the support of the OSCE [40,41].

In our 2005 report we noted that since 2000, when an annual survey concerning events in 1998 was completed, the authorities had not reported any seizures of opiates or chemicals, although significant quantities had been seized in previous years [11]. This has changed in the meantime. Turkmenistan has again started to report to the relevant UN agency, notifying seizures up to 2005 [42,43].

Under Niyazov, however, the existence of drug problems in the country was denied. What is equally worrying is the absence or inadequacy of measures addressed to meet the needs of drug users and other vulnerable populations. Many of the HIV prevention projects for drug users and sex workers initiated by the United Nations Development Programme (UNDP), USAID and the Open Society Institute have ground to a halt.

One of the main reasons for increased drug use is the greater availability of drugs. Turkmenistan shares more than 700 km of poorly policed border with Afghanistan, the largest producer of opium in the world. At least 25–30% of narcotics produced in Afghanistan are currently transiting Central Asia on their way to Russian and Western European markets [35]. Afghan opiates are not only smuggled through Turkmenistan by land (in trucks and cars), but also by sea (on vessels passing through the Caspian Sea) and by air (on cargo planes bound for Azerbaijan and Turkey) [44].

While drug use has been a traditional phenomenon in Turkmenistan, the users were mainly elderly men using marijuana and opium. After independence, heroin became much more prevalent, used by younger people and also linked to sex work [37,45], reflecting a shift occurring throughout Central Asia [46]. Apart from the large-scale trafficking of opium and heroin from Afghanistan, the alarming increase in drug use in Turkmenistan in recent years has also been fuelled by economic hardship, corruption, and an increase in sex work and crime [21,28,35].

The extent of drug use in the country can only be estimated. The number of drug users officially registered by the Ministry of Health and Medical Industry increased massively, from 3,704 in 1989 to 43,947 in 2003 [47], with about 20% of drug users injecting drugs [34,48]. However, much as in the rest of Central Asia, the limited quality and accessibility of drug treatment services, combined with the stigmatisation of drug users and official registration by the police, mean that many drug users are unwilling to seek treatment and are not recorded in official statistics [35,45]. Unsafe injecting practices are widespread and harm reduction measures largely absent, contributing to the spread of communicable diseases, including HIV/AIDS [34,48]. A former prisoner reported that " [h]alf the prisoners are drug addicts, and syringes are passed from one person to the next" [21].

UNODC estimated that, throughout Central Asia, a 17-fold increase of opiate use occurred in the period 1990–2002. Approximately 1% of the total population of the region are estimated to be injecting drug users [35]. It has also been estimated that up to 20,000 people in Ashgabat alone are involved in the drugs trade [37] and that 70% of young sex workers in Ashgabat are addicted to heroin [49]. One university student observed that "I've yet to reach my 20th birthday, but already half of my classmates and neighbours – the friends I grew up with – are seasoned drug addicts" [37]. Reportedly, some young people now offer heroin at weddings [34,37]. In 2005, it was reported that one can buy a dose of heroin in Ashgabat for less than a couple of dollars [50].

In his election campaign, the new president Berdymukhammedov raised the hitherto taboo topic of drug abuse and described it as "a misfortune for the whole of mankind" [51]. He promised to eradicate the trafficking of drugs from Afghanistan and to fight widespread drug addiction [52]. It remains to be seen whether he will be able and willing to live up to these promises.

### The neglect of the health care system

Article 12 of the International Covenant on Economic, Social and Cultural Rights confers on member states the responsibility to "assure to all medical service and medical attention in the event of sickness" [17]. While health care is only one factor among many others impacting on population health, it remains crucial to ensure health and well-being [13]. In Turkmenistan, the absence of democracy and human rights has meant that the public has not been involved in health policy development, so that its views were not taken into consideration. Although the Soviet health system had a number of inherent flaws, it provided comprehensive medical care free at the point of delivery. Since the independence of Turkmenistan in 1991, funding for health care has declined and health services have deteriorated, with shortages of medicines and medical equipment. Years of underinvestment in the almost entirely state-owned and managed health system have led to deteriorating buildings and equipment [53-55].

While these are problems that face, to different degrees, all health systems in Central Asia, the situation in Turkmenistan is peculiar, in that no comprehensive health reform has been undertaken in recent years and the restructuring of the health sector has been based on *ad hoc *decisions of the president and the attempt to cut governmental health financing. In addition, there is no transparent state budget for the health sector, so that it is impossible to establish the share of resources being spent on health.

In January 2004, the president signed a decree envisaging the dismissal of 15,000 health care workers (including doctors, nurses, midwives, medical attendants and orderlies), to take effect on 1 March 2004. This affected an estimated one-third of the medical workforce, who were to be replaced by unqualified military conscripts, again a measure unparalleled in Central Asia [56-60]. The decree declared that the dismissal would encourage the "effective use of [remaining] medical personnel and the transition to a system of private or partly self-financing health care" [60]. The move formed the latest in a series of cuts to the country's health care system [61]. It aimed to "cover up a large deficit in the government budget" [62], attributed in part to corruption [63]. In 2001, the number of state-employed health care workers had already been cut by several thousands to save money [64].

One midwife in Ashgabat justifiably asked: "will young people lacking specialist education really be able to help deliver babies or give injections?" [60]. While those dismissed were expected not to complain [56], a nurse noted that "[t]hey usually fire women who have a poor sick-leave record – normally mothers with young children who are often ill and need attention" [65]. There is no systematic research into the effects on those loosing their jobs, but there have been a number of personal accounts, such as that of a nurse who reportedly committed suicide after being dismissed [65]. Elena, a former health care worker interviewed by the Institute for War and Peace Reporting (IWPR), is a trained doctor, but was required to work as a nurse in recent years and then even lost this job when hospital staff was replaced with conscript soldiers. Her son took to begging on the streets [66]. Another former nurse disclosed to undercover BBC reporters that she was forced to turn to prostitution [67].

The decree of January 2004 also introduced user fees for an increased range of medical services as of 1 March 2004 [57,68]. As in other countries in the former Soviet Union, user fees had been introduced incrementally since independence. In 1998, they were extended to include self-referred patients, some diagnostic procedures and consultations, cosmetic surgery, dental care and physical therapies [54,69]. Medical facilities specialising in ophthalmic, dental, skin, gastrointestinal and cardiovascular diseases charged fees for their services from March 2004 onwards [70]. While details of possible exemptions to fees are currently unclear, this can be expected to increase inequality and impoverishment if families have to bear the cost of illness. In 2005, however, president Niyazov announced a significant reduction in these fees [9].

Grandiose presidential projects are being pursued at the same time. Just after deciding to lay off the 15,000 health care professionals, Niyazov announced the construction of a new building for the Ministry of Health and Medical Industry at a cost of US$12 million, allocated from the budget of the Ministry of Health and Medical Industry. The building was to be constructed in the shape of the snake that symbolizes medicine [71].

Although recent data are lacking, even by the end of the 1990s it was clear that access to health services had deteriorated dramatically. Health care workers were being paid irregularly and informal payments were widespread [53-55,72]. According to a Turkmen medical practitioner, " [a] person will be dying at home and not go to a hospital because he cannot afford to pay for his treatment" [19,56]. At the beginning of October 2004, Niyazov announced that 3,000 draftees would be sent to medical centres, although it was not clear if this would entail further dismissals of health care workers [73].

In February 2005, Niyazov ordered the closure of all hospitals (and public libraries) outside Ashgabat and all but one diagnostic centre in each oblast centre [14,74,75]. However, following protests by the international community, the order was not comprehensively implemented.

Despite this overall depressing backdrop, some initiatives supported by international agencies have yielded positive results. One of them is that universal salt iodization has made Turkmenistan the first country in Central Asia and fourth in the world to achieve optimum iodine nutrition [9]. In addition, flour fortification takes place in 17 of Turkmenistan's 18 largest mills [9]. Immunization coverage rates generally remained high and the country was certified as polio-free in 2002 [9]. These developments have led UNICEF to conclude that Turkmenistan "has had success in improving maternal and child health" [9]. However, this conclusion is not supported by UN estimates of life expectancy or infant mortality.

There is a general agreement that health services have deteriorated in recent years, with a shortage of qualified staff, especially at primary care level, and a shortage of essential drugs and medical supplies [9]. The World Bank country brief of 2006 observed that "the quality of education and basic health services has [...] significantly deteriorated over the past ten years" [8]. In this context, the United Nations Development Assistance Framework (UNDAF) for 2005–2009 has made the achievement of "user-friendly and sustainable health care and nutrition services [...] in compliance with international standards at the national and sub-national levels" [76] one of its aims.

Following publication of our 2005 report, two courageous BBC reporters, Lucy Ash and Sian Glaessner, visiting Turkmenistan incognito, confirmed the devastating consequences of Niyazov's policies, illustrated by an interview with a nurse who, like many of her colleagues, had lost her job and was forced to turn to prostitution [67]. Some ordinary Turkmens reportedly say they are reluctant to take their children to doctors who graduated from Turkmen medical institutions during Niyazov's rule [77].

Most candidates for the presidential elections in February 2007 acknowledged that something needed to be done about the parlous state of Turkmenistan's health and education systems [77]. The new president, Berdymukhamedov, has promised to improve health services, with modern facilities, a new institute to train doctors and greater access to Western medicine [77].

### What is the extent of the current health crisis?

In Turkmenistan, health indicators such as life expectancy and infant mortality were historically among the worst in the former Soviet Union countries [8]. The last year for which Turkmenistan reported most mortality statistics to the WHO Regional Office for Europe was 1998. Officially reported life expectancy of 66.1 years in 1998 was lower than in all other Central Asian countries, when considering the most recent data for all countries [78]. More up-to-date data on life expectancy have been reported by the country to the MONEE database of UNICEF. According to these data, in 2004 male life expectancy reached 66.1 years and female life expectancy 72.2 [26].

Yet, estimates of life expectancy in Turkmenistan by international organisations are considerably lower. Although the ways in which these estimates have been calculated are in no way clear [79], most seem to take some account of the underestimation of infant mortality in official data [80]. Using the WHO estimate of life expectancy from the World Health Report, Turkmenistan, at 60 years in 2003, had the lowest value in any country in Europe and Central Asia, almost 20 years lower than the EU-15 average [78]. All other Central Asian countries achieved higher life expectancies, although most of them have a much lower real gross domestic product per capita. According to World Bank estimates, in 2004 life expectancy in Turkmenistan was 67.06 for females, 58.55 for males and 62.70 years for both sexes [10].

Turkmenistan is among the 50 countries with the highest under-five mortality rates, according to the UNICEF State of the World's Children 2007 report. Infant mortality, officially registered at 14.1 per 1,000 live births [26], was estimated by UNICEF at 81 per 1,000 live births in 2005 [9], many times higher than in advanced industrialised countries.

According to official data, maternal mortality stood at 16.77 per 100,000 live births in 2004 [78]. This is almost certainly an underestimate. UN organisations estimated the real maternal mortality rate at 31 per 100,000 live births in 2000 [78]. The Demographic and Health Survey in 2000 showed that 47% of women of reproductive age (15–49 years) and 36% of children under 5 years have anaemia [9].

## Conclusion

Have the human rights violations in Turkmenistan contributed to the health crisis experienced by the country? Focusing on three areas where the implications are most apparent, our paper strongly suggests that this is the case.

Although Turkmenistan has sought international assistance in the fight against HIV/AIDS and the spread of avian influenza and has also reported some health data to the international community, it still seems to cling to the previous policy of secrecy and denial. The data on HIV/AIDS, which fail to capture a single case in the country in recent years, are the most obvious example for this policy. The denial of health problems in the country is likely to lead to their persistence or escalation.

In terms of drug trafficking, the true extent of the involvement of the Niyazov regime in the drug trade from Afghanistan may only now become apparent. The new president Berdymukhammedov raised the previously taboo topic of drug abuse in his election campaign and promised to eradicate the trafficking of drugs from Afghanistan. Only time will tell whether he can muster the political support in the higher echelons of the regime to follow these words with actions.

The worrying decline in health services in Turkmenistan in the last years of the Niyazov regime has been noted by several international agencies. It has also become a topic in the presidential election campaigns in early 2007 and Berdymukhammedov promised to invest in the health sector. As he has been Minister of Health in the crucial period, however, these promises have to be treated with some scepticism.

Despite the abject record of Niyazov's regime, its plentiful energy resources and collaboration in the so-called "war on terror" meant that critique by the major powers was muted. After we published our report in 2005, we briefed the main UN agencies, the European Union, the UK Foreign and Commonwealth Office and the US State Department, and although there was much support by desk staff, politics prevailed.

After Niyazov's death, the main concern of the major powers was one of regional stability and secure energy supply, and human rights concerns once again played a secondary role. The strategic importance of the inauguration of Berdymukhamedov as Turkmenistan's new president in February 2007 was underscored by the presence of Russian Prime Minister Mikhail Fradkov and US Assistant Secretary of State Richard Boucher, as well as senior delegations from Iran and China. Human rights groups have argued that the limited pledges of the new president are not enough, and that instead of giving the new leadership the benefit of the doubt, the international community should be pressing for change [51]. Indeed, Berdymukhammedov has emphasized repeatedly that he would continue the policies of Niyazov [81]. Beginning the post-Niyazov era with a blatantly falsified election is certainly not an encouraging sign.

## Competing interests

The author(s) declare that they have no competing interests.

## Authors' contributions

BR participated in the design of the study, performed the data collection, and drafted the manuscript. MM conceived of the study, participated in its design and coordination, and helped to draft the manuscript. All authors read and approved the final manuscript.

## Pre-publication history

The pre-publication history for this paper can be accessed here:



## References

[B1] BBC News New Turkmen Leader is Inaugurated, 14 February 2007. http://news.bbc.co.uk/1/hi/world/asia-pacific/6359569.stm.

[B2] The Guardian Turkmenistan Votes to Replace Dictator, 12 February 2007. http://www.guardian.co.uk/international/story/0,,2010923,00.html.

[B3] Human Rights Watch World Report 2007. http://hrw.org/wr2k7/#nolink.

[B4] Reporters Without Borders Worldwide Press Freedom Index 2006. http://www.rsf.org/rubrique.php3?id_rubrique=639.

[B5] US Department of State Turkmenistan. Country Reports on Human Rights Practices, 2005. http://www.state.gov/g/drl/rls/hrrpt/2005/61681.htm.

[B6] IRIN Prison Conditions Remain Bleak in Turkmenistan, 30 September 2004. http://www.irinnews.org/report.asp?ReportID=43435&SelectRegion=Central_Asia&SelectCountry=TURKMENISTAN.

[B7] USAID Country profile: Turkmenistan, August 2005. http://turkmenistan.usembassy.gov/uploads/gd/Bw/gdBw1XfCC7kF5L-E-jcUIw/USAIDTurkmenistan-Profile_8-05.pdf.

[B8] World Bank Turkmenistan Country Brief, updated September 2006. http://web.worldbank.org/WBSITE/EXTERNAL/COUNTRIES/ECAEXT/TURKMENISTANEXTN/0,,menuPK:300741~pagePK:141159~piPK:141110~theSitePK:300736,00.html#Economy.

[B9] UNICEF Turkmenistan Country Website. http://www.unicef.org/infobycountry/Turkmenistan_466.html.

[B10] World Bank (2006). World Development Indicators.

[B11] Rechel B, McKee M Human Rights and Health in Turkmenistan. http://www.lshtm.ac.uk/ecohost/projects/turkmenistan%20files/Turkmen%20report.pdf.

[B12] Franco A, Alvarez-Dardet C, Ruiz MT (2004). Effect of democracy on health: ecological study. BMJ.

[B13] Mann JM, Gostin L, Gruskin S, Brennan T, Lazzarini Z, Fineberg H, Mann JM, Gruskin S, Grodin MA, Annas GJ (1999). Health and human rights. Health and Human Rights: a Reader.

[B14] UN Universal Declaration of Human Rights. http://www.un.org/Overview/rights.html.

[B15] UN Resident Coordinator (2006). UN Resident Coordinator's Report for Turkmenistan 2005.

[B16] USAID Turkmenistan Converts to WHO Live Birth Standard. http://www.usaid.gov/locations/europe_eurasia/press/success/2007-02-24.html.

[B17] UN International Covenant on Economic, Social and Cultural Rights. http://www.unhchr.ch/html/menu3/b/a_cescr.htm.

[B18] BBC Turkmen Medics Told Not to Diagnose "Banned" Diseases, 24 May 2004. http://www.watan.ru/eng/view.php?nomer=460&razd=new_nov_en&pg=57.

[B19] Eurasianet Eurasia Insight: Reported Plague Outbreak Renews Concerns About Turkmenistan's Healthcare System, 19 July 2004. http://www.eurasianet.org/departments/insight/articles/eav071904.shtml.

[B20] Turkmenistan Project Certain Diseases "Banned" in Turkmenistan, Press release No. 43, 8 May 2004. http://www.eurasianet.org/turkmenistan.project/index.php?page=wnb/wnb040521&lang=eng.

[B21] Institute for War and Peace Reporting Turkmenistan: Women Drawn into Drug Trade, 12 August 2005. http://iwpr.net/?p=rca&s=f&o=255884&apc_state=henirca2005.

[B22] RadioFreeEurope/RadioLiberty Turkmenistan: Hidden Resurgence Of Plague Threatens, 30 June 2004. http://www.rferl.org/featuresarticle/2004/06/bdb66fd4-10ea-4bd4-9145-d9abbb3461aa.html.

[B23] Institute for War and Peace Reporting Turkmen Doctors Fear Epidemic, 13 July 2004. http://iwpr.net/?p=rca&s=f&o=175546&apc_state=henirca2004.

[B24] Los Angeles Times Turkmenistan: An "Illegal" Outbreak of Plague, 8 August 2004. http://www.watan.ru/eng/view.php?nomer=552&razd=new_nov_en&pg=53.

[B25] UNAIDS Turkmenistan. 2006 update. Epidemiological Fact Sheets on HIV/AIDS and Sexually Transmitted Infections. http://www.who.int/globalatlas/predefinedReports/EFS2006/index.asp.

[B26] UNICEF TransMonee Project Database 2006. http://www.unicef-irc.org/databases/transmonee/.

[B27] Bernitz BL, Rechel B, Matic S, Lazarus J, Donoghoe MC (2006). HIV data in Central and Eastern Europe: Fact or Fiction?. HIV/AIDS in Europe: Moving from Death Sentence to Chronic Disease Management.

[B28] Turkmenistan. http://www.unaids.org/en/Regions_Countries/Countries/turkmenistan.asp.

[B29] Godinho J, Novotny T, Tadesse H, Vinokur A (2004). HIV/AIDS and Tuberculosis in Central Asia, Country Profiles.

[B30] USAID (2003). Final Report on HIV/AIDS Prevention Projects in Turkmenistan, October 2002 to November 2003.

[B31] USAID Turkmenistan Seeks Global Fund Aid to Fight Health Problems. http://www.usaid.gov/locations/europe_eurasia/press/success/2006-10-20.html.

[B32] USAID Turkmenistan Launches First Rural TB-DOTS Pilot Site. http://www.usaid.gov/locations/europe_eurasia/press/success/2006-12-17.html.

[B33] Institute for War and Peace Reporting Avian Flu Restrictions Still in Place, 4 February 2006. http://iwpr.net/?p=trk&s=f&o=326273&apc_state=henptrk.

[B34] IRIN Turkmenistan: Heroin Use Poses a Growing Challenge, 5 October 2004. http://www.irinnews.org/report.aspx?reportid=25851.

[B35] UNODC (2004). Strategic Programme Framework, Central Asia, 2004–2007, June 2004.

[B36] Freedom House Nations in Transit 2004: Turkmenistan. http://www.freedomhouse.org/template.cfm?page=47&nit=346&year=2004.

[B37] Institute for War and Peace Reporting Turkmenistan's Rising Drugs Crisis, 22 June 2004. http://iwpr.net/?p=rca&s=f&o=175878&apc_state=henirca2004.

[B38] Annagurban Y Turkmenistan: Sultanistic State. http://www.gundogar.org/?0220041408000000000000011000000.

[B39] UN Press Release SOC/NAR/891: Narcotics Control Board Concerned about Failure of Turkmenistan to Cooperate With International Community in Fight Against Illicit Drugs, 3 March 2004. http://www.un.org/News/Press/docs/2004/socnar891.doc.htm.

[B40] UNODC (2006). Milestones Issue 3, April 2006.

[B41] RadioFreeEurope/RadioLiberty UN, OSCE Bolster Turkmenistan's Anti-Trafficking Battle, 15 February 2006. http://www.rferl.org/featuresarticle/2006/02/940c9d8a-fa0f-44b1-84fd-f5f841775187.html.

[B42] UNODC (2006). 2006 World Drug Report Volume 1: Analysis.

[B43] UNODC Turkmenistan country fact sheet. http://www.unodc.org/uzbekistan/en/fact_tuk.html?print=yes.

[B44] Fenopetov V (2006). The drug crime threat to countries located on the 'silk road'. China Eurasia Forum Quarterly.

[B45] Kerimi N (2000). Opium use in Turkmenistan: a historical perspective. Addiction.

[B46] UNODC (2002). Illicit Drugs Situation in the Regions Neighbouring Afghanistan and the Response of the ODC, November 2002.

[B47] UNICEF TransMonee project database 2004.

[B48] UNICEF At a Glance: Turkmenistan. http://www.unicef.org/infobycountry/Turkmenistan.html.

[B49] Institute for War and Peace Reporting Turkmenistan: Poverty Drives Addiction and Prostitution, 3 September 2004. http://iwpr.net/?apc_state=hruirca2004&l=en&s=f&o=174844.

[B50] IRIN Turkmenistan: Drug addiction on the Rise, 2 August 2005. http://www.irinnews.org/report.asp?ReportID=48406&SelectRegion=Asia&SelectCountry=TURKMENISTAN.

[B51] IRIN Turkmenistan: President Sworn in Amid Cautious Hope for Change, 14 February 2007. http://www.irinnews.org/report.aspx?ReportId=70173.

[B52] International Herald Tribune Turkmen officials destroy 562 kilograms of illegal drugs, 7 February 2007. http://www.iht.com/articles/ap/2007/02/07/asia/AS-GEN-Turkmenistan-Drugs.php.

[B53] WHO (2000). Highlights on Health in Turkmenistan.

[B54] Mamedkuliev C, Shevkun E, Hajioff S (2000). Health Care Systems in Transition: Turkmenistan.

[B55] Mamedkuliev C, Shevkun E, Hajioff S, McKee M, Healy J, Falkingham J (2002). Turkmenistan. Health Care in Central Asia.

[B56] BBC Large-scale layoff of medical workers planned in Turkmenistan, 21 January 2004. http://www.watan.ru/eng/view.php?nomer=290&razd=new_nov_en&pg=60.

[B57] BBC Troops to replace Turkmen medics, 1 March 2004. http://news.bbc.co.uk/1/hi/world/asia-pacific/3522855.stm.

[B58] The Observer The Man Who Would Be King, 10 October 2004. http://observer.guardian.co.uk/magazine/story/0,,1323246,00.html.

[B59] The Times And the Prize for the Greatest Megalomaniac in the World Goes to... You Know the Name, 31 May 2004. http://www.watan.ru/eng/view.php?nomer=468&razd=new_nov_en&pg=57.

[B60] Reuters Turkmen President Sacks 15,000 Nurses, 10 February 2004.

[B61] RadioFreeEurope/RadioLiberty Turkmenistan: Flag Day Marked as President Prepares to Show 'Real Turkmenistan' to the World. http://www.rferl.org/featuresarticle/2004/02/af2cac52-8eb8-4d9b-912f-22d755c9c7f5.html.

[B62] Institute for War and Peace Reporting Turkmenistan: No to Foreign Education, 18 May 2004. http://iwpr.net/?p=rca&s=f&o=176269&apc_state=henirca2004.

[B63] Institute for War and Peace Reporting Turkmen Troops Double Up as Nurses and Bakers, 25 February 2004. http://iwpr.net/?p=rca&s=f&o=177249&apc_state=henirca2004.

[B64] RadioFreeEurope/RadioLiberty News Briefs From and About Turkmenistan, 12 January 2004. http://www.rferl.org/reports/turkmen-report/2004/01/0-120104.asp.

[B65] Institute for War and Peace Reporting Turkmen Nurses Devastated by Decree, 5 March 2004. http://iwpr.net/?p=rca&s=f&o=176975&apc_state=henirca2004.

[B66] Institute for War and Peace Reporting Turkmenistan: Reduced to Begging, 11 May 2004. http://iwpr.net/?p=rca&s=f&o=176294&apc_state=henirca2004.

[B67] BBC World Service Undercover in Turkmenistan: the First of Four New World Service Investigations, 9 November 2005. http://www.bbc.co.uk/pressoffice/pressreleases/stories/2005/11_november/09/turkmenistan.shtml.

[B68] Institute for War and Peace Reporting Turkmen Patients Pay for Privatisation, 26 April 2005. http://www.watan.ru/eng/view.php?nomer=852&razd=new_nov_en&pg=38.

[B69] Healy J, Falkingham J, McKee M, McKee M, Healy J, Falkingham J (2002). Health care systems in transition. Health Care in Central Asia.

[B70] Kurbanova A Turkmenistan to introduce paid medical services. ITAR-TASS, 8 January 2004.

[B71] Turkmenistan.ru Turetskaia kompania postroit v Ashgabade novoe zdanie Minizdrava stoimustiu 12 min. doll. SShA [Turkish company to build new US$12 million building for the Ministry of Health in Ashgabat]. Turkmenistan ru, 7 April 2004.

[B72] Ensor T, Amannyazova B (2000). Use of business planning methods to monitor global health budgets in Turkmenistan. Bulletin World Health Organization.

[B73] BBC More Turkmen Conscripts to be Offered Alternative Service. http://www.watan.ru/eng/view.php?nomer=80&razd=new_eko_en&pg=11.

[B74] CERD Concluding Observations of the Committee on the Elimination of Racial Discrimination: Turkmenistan, 2005. http://www.unhchr.ch/tbs/doc.nsf/898586b1dc7b4043c1256a450044f331/7d24c9f7d03cf167c12570b20036511d/$FILE/G0741016.pdf.

[B75] Institute for War and Peace Reporting Turkmenbashi Wields The Axe, 11 March 2005. http://iwpr.net/?p=rca&s=f&o=238797&apc_state=henirca2005.

[B76] United Nations Country Team (2004). United Nations Development Assistance Framework (UNDAF) 2005–2009. Ashgabat.

[B77] IRIN Turkmenistan: Election Pledges Raise Hope of Change. http://www.irinnews.org/report.asp?ReportID=57558.

[B78] WHO Health for All Database, January 2007. http://www.euro.who.int/hfadb.

[B79] Rechel B, Shapo L, McKee M (2004). Millennium Development Goals for Health in Europe and Central Asia Relevance and Policy Implications – World Bank Working Paper No 33.

[B80] WHO (2006). Highlights on Health in Turkmenistan 2005.

[B81] Human Rights Watch Turkmenistan: No Deals Without Rights Reform. http://hrw.org/english/docs/2007/02/08/turkme15285.htm.

